# FcRn Overexpression Expands Diversity of the Humoral Immune Response in bFcRn Transgenic Mice

**DOI:** 10.3389/fimmu.2020.01887

**Published:** 2020-08-21

**Authors:** Bence Szikora, Anita Marx, Péter K. Jani, Orsolya Pipek, Viktor Müller, István Csabai, Imre Kacskovics

**Affiliations:** ^1^Department of Immunology, Institute of Biology, Eötvös Loránd University, Budapest, Hungary; ^2^ImmunoGenes Ltd., Budakeszi, Hungary; ^3^Department of Physics of Complex Systems, Institute of Physics, Eötvös Loránd University, Budapest, Hungary; ^4^Department of Plant Systematics, Ecology and Theoretical Biology, Institute of Biology, Eötvös Loránd University, Budapest, Hungary

**Keywords:** B cell repertoire, next generation sequencing, FcRn overexpression, humoral immune response, monoclonal antibody production

## Abstract

The neonatal Fc receptor (FcRn) plays key roles in IgG and albumin homeostasis, maternal IgG transport, and antigen presentation of IgG-opsonized antigens. Previously, we reported that transgenic (Tg) mice that overexpress bovine FcRn (bFcRn) have augmented T-dependent humoral immune response with increased IgG protection, higher level of antigen-specific antibodies, greater number of antigen-specific B cells, and effective immune response even against weakly immunogenic epitopes. In this study we analyzed the diversity of the humoral immune response of bFcRn Tg mice, using a length distribution analysis (spectratyping) and next generation sequencing (NGS) of the immunoglobulin heavy chain variable regions. Our analysis showed that in response to immunization with ovalbumin or transfected cells that expressed a unique membrane protein, our Tg animals developed a more diverse plasma cell repertoire than controls, which manifested in greater numbers of different clones, and clusters with fewer highly expanded large clones, as identified by the variable region (CDR3) of the immunoglobulin heavy chain. The increased antibody diversity in Tg mice after immunization was observed at both IgM and IgG levels, indicating that the increased humoral immune diversity in Tg mice is due to a higher number of both activated, antigen-specific naïve and isotype switched B cells. We thus demonstrated that the BCR repertoire of the immunized bFcRn Tg animals is more diverse compared to wild type mice, which likely makes these Tg mice a better choice for monoclonal antibody production against challenging antigens, including the extracellular regions of cell membrane proteins.

## Introduction

In recent years, there has been an increasing demand for the development of faster and more efficient technologies to produce high-affinity monoclonal antibodies (mAb) against specific epitopes of targets that are weakly immunogenic or even tolerogenic. One of these technologies is to use genetically modified mice with enhanced humoral immune response.

Amongst the receptors for Fc portion (FcRs) of immunoglobulin (Ig) molecules, FcRn is one of the key players that regulates IgG homeostasis and was originally identified as the protein that regulates IgG catabolism (1) and mediates maternal IgG transport (2, 3). FcRn was shown to bind IgG in a strictly pH-dependent manner, with binding occurring at slightly acidic pH (4, 5), and it is composed of a major histocompatibility complex (MHC) class I-like α-chain and the β2-microglobulin (B2M) (6). Similarly to other FcRs (PMID: 31130948, PMID: 11244038, PMID: 10586892, PMID: 2140512, PMID: 12524384), FcRn was also found to have important impacts on immune homeostasis in different ways. It has a dominant role in regulating the transport of IgG within and across cells of diverse origins, and it also serves to rescue IgG and albumin in capillary endothelial and hematopoietic cells from degradation, prolonging their half-lives (7, 8). In addition, FcRn was demonstrated to facilitate antigen (Ag) presentation in case of Ag–IgG immune complexes (IC) by professional antigen-presenting cells (APCs) stimulating MHC class II and also MHC class I-related T-cell activation (9).

We have previously shown that overexpression of the FcRn in transgenic (Tg) mice and rabbits extended IgG half-life (10, 11). These mice also have augmented T dependent humoral immune response, which manifests in higher antigen specific antibody titers, greater number of antigen specific, activated T helper cells, increased number of activated antigen specific B cells, bigger spleen, and increased size and numbers of germinal centers in the spleen after intraperitoneal immunization (12–16). A larger pool of antigen specific B cells facilitates monoclonal antibody (mAb) discovery as it allows more effective identification of the appropriate B-cell clones either using standard hybridoma or novel high throughput technologies. In this study we sought to elucidate whether the increased number of antigen specific B cells in these Tg animals was simply the result of a greater expansion of the same number of originally activated B cells, or it also reflected an increased variability among naïve B cells generated during an immune response. Obviously, greater diversity would further aid mAb discovery by allowing the identification of mAbs that are specific for unique epitopes and/or have unique characteristics.

Using a microarray-based oligopeptide scanning study, we have previously demonstrated that ovalbumin immunization results in a greater breadth of immune response (as tested on a large array of oligopeptides) in the Tg mice compared with wild type controls (14). These results, and the fact that the Tg animals that overexpress FcRn were able to produce high quality antibodies against weakly immunogenic and very challenging antigens suggested that they have an increased diversity of B-cell repertoire (17, 18).

Diversity in the primary antibody repertoire (before exogenous antigen exposure) stems from the allelic diversity in immunoglobulin gene segments, combinatorial diversity introduced during somatic recombination, junctional diversity caused by the imprecision of the recombination process, pairing of IgH and IgL polypeptide chains, and receptor editing, wherein the existing V-gene segment is replaced with another. Most of the diversity in the naïve antibody repertoire is concentrated at the site of IgH VDJ gene segment junction, also known as the IgH complementarity-determining region 3 (CDR-H3). Diversification of the post-antigen-stimulation secondary antibody repertoire stems from somatic hypermutation and class-switch recombination (19).

There are many different techniques which can be used for analyzing the B-cell repertoire at nucleotide level (20). CDR3 spectratyping reveals the length distribution of the regions: it cannot distinguish between individual sequences, but it can provide a quick insight into the diversity of the repertoire (21). In this study, we used a similar approach, where we analyzed the length of the whole variable region, not just the CDR3, to get an insight into the diversity of the antibody repertoire. More recently, next generation sequencing (NGS) has become a powerful and sensitive tool for analyzing antibody repertoire diversity (19, 22, 23). Using appropriate protocols, error correction pipelines (24), and bioinformatic evaluation programs, the Ig-seq (high-throughput DNA sequencing of immunoglobulin genes) can provide a broad, deep and correct picture of the antibody repertoire (25). To study the diversity of the humoral immune response of Tg mice, we first analyzed the length distribution of the variable regions of immunized wild type (wt) and Tg mice by spectratyping. Then, we compared the antibody repertoires of Tg and wt mice before and after immunization with different antigens, by performing Ig-seq of both IgG and IgM repertoires.

## Materials and Methods

### Animals

We used hemizygous Tg mice that carry five copies of the bFcRn α-chain encoding gene (bovine FCGRT) in addition to the endogenous mouse FCGRT gene on BALB/c genetic background [BALB/c_Tg5_Bfcgrt] (12). Wt BALB/c mice were littermates of the Tg animals born from hemizygous breeding. Mice were kept under specified pathogen free (SPF) conditions in individual ventilation cages (IVC) in the animal house of the Department of Immunology, Eötvös Loránd University, Budapest, Hungary.

### Ethics Statement

Experiments on mice were carried out in strict accordance with the recommendations of the Guide of the Institutional Animal Care and Ethics Committee at Eötvös Loránd University, in accordance with permissions PEI/001/2196-2/2013 issued by the Food Chain Safety and Animal Health Directorate of the Government Office of Pest County, Hungary.

### Mouse Immunization

Different immunization protocols were used for each experiment (Table 1). Briefly, 7–8 weeks old female wt or Tg mice (minimum of four in each group) were immunized either with ovalbumin (OVA, Sigma), 3T3-ABCC1 or HEK-ABCC1 cells (i.e., 3T3 or HEK cells stably transfected with human multidrug resistance-associated protein 1 encoded by the ABCC1 gene), provided by András Várady, Hungarian Academy of Sciences, Institute of Enzymology. In case of OVA immunizations, the animals were intraperitoneally (i.p.) immunized with 50 μg OVA in complete Freund adjuvant (CFA) (1:1, Sigma) and boosted 21 days later with 25 μg OVA in incomplete Freund adjuvant (1:1, Sigma). Animals were sacrificed on day 24 for NGS or length distribution analysis. In case of cellular immunizations (3T3-ABCC1, HEK-ABCC1) the mice were i.p. immunized with 5^*^10E6 cells in CFA (1:1) and boosted 14, 28, and 63 days later with 5^*^10E6 cells in IFA (1:1). Sera were analyzed by flow cytometry (FCM) on day 21 and 35 to detect ABCC1 specific immune response, and animals were sacrificed on day 66 for NGS. 10–11 weeks old female mice were used to obtain the non-immunized samples.

**Table 1:** Immunization protocols with different antigens.

**Time**	**Ovalbumin**	**HEK-ABCC1**	**3T3-ABCC1**
Day 0	50 μg OVA +CFA	5*10E6 cells + CFA	5*10E6 cells + CFA
Day 14		5*10E6 cells + IFA	5*10E6 cells + IFA
Day 21	25 μg OVA + IFA		
Day 24	NGS or length distribution analysis		
Day 28		5*10E6 cells + IFA	5*10E6 cells + IFA
Day 35		FCM analysis	FCM analysis
Day 63		5*10E6 cells + IFA	5*10E6 cells + IFA
Day 66		NGS analysis	NGS analysis

### Cell Isolation and Sorting

For the analysis of the B-cell repertoire the spleen was isolated from the animals and was disintegrated using syringe in GKN (0.4 g/l KCl, 8 g/l NaCl, 1.77 g/l Na_2_HPO_4_·2H_2_O, 0.69 g/l NaH_2_PO_4_ H_2_O, 2 g/l glucose, 10 mg/l phenol red). Red blood cells were lysed for 90 s in ACK (8.3 g/l NH_4_Cl, 1 g/l KHCO_3_, 0.038 g/l Na_2_EDTA, pH 7.3) solution, then the rest of the cells were filtered through a cell strainer (70 μm, Bd Falcon). The cells were counted by Bürker chamber, and the concentration was adjusted to 10E8 cell/ml in FACS buffer (PBS, 1% FCS, 0.1% Na-azide). 0.5 μg/mL PE-conjugated anti-CD138 antibody (BD Pharmingen) was added to the cell suspension on ice, for 25 min. The cell suspension was then washed twice with 3–3 ml FACS buffer. Finally, cells were resuspended in FACS buffer in 5^*^10E7 cell/ml concentration for sorting. The CD138^+^ cells were sorted out using FACS ARIA III (BD), and were subsequently lysed in lysis buffer (RNeasy plus mini kit, Qiagen) for RNA isolation.

For the analysis of the OVA-specific B-cell repertoire the similarly prepared splenocytes were incubated with Alexa488 conjugated (Invitrogen) OVA (5 μg) and PerCP-Cy5.5 labeled anti-CD19 antibody (BD Pharmingen) for 25 min.

The whole spleen was prepared unless indicated otherwise.

### ELISA Measurements of the OVA Specific Antibody Titer

High-binding ELISA plates (Costar 9018, Corning, NY) were coated with OVA (2 μg/ml, Sigma-Aldrich) in 0.1 M sodium carbonate–bicarbonate buffer (pH 9.6) for 2 h at room temperature and then were washed with 0.1 M PBS (pH 7.2) containing 0.05% Tween 20 (PBS-Tween). Serially diluted serum samples were added to the wells (1:5 serial dilution beginning from 1:1,000) and incubated for 1 h at room temperature. After washing again with PBS-Tween, bound serum Ab was revealed by HRP-labeled goat anti-mouse IgG (1:5,000-fold dilution, Southern Biotechnology Associates). The peroxidase-conjugated Abs were detected using tetramethylbenzidine (Sigma-Aldrich) as the substrate, and OD at 450 nm was measured with the Multiscan ELISA Plate Reader (Thermo EC). Serial dilutions of each serum sample were applied, and OVA specific IgG titers as end-point titers were determined by GraphPad Prism 5 non-linear regression to the hyperbolic saturation function (end-point titer: the dilution factor of the sera where the OD value is 0.1).

### Length Distribution Analysis of the Heavy Chain Variable Regions

Total RNA was isolated from CD138^+^ splenocytes of wt and Tg animals (Table 2) using RNeasy plus mini kit (Qiagen) according to the manufacturer's protocol. RNA concentration was measured on a Nanodrop ND-1000 spectrophotometer. Next, IgG-specific cDNA of total mRNA was prepared with 12.5 μl of total RNA using the following conditions: Maxima reverse Transcriptase (Thermo Fisher Scientific, 200 U), Ribolock RNAse inhibitor (20 U), 5^*^RT buffer, dNTP mix (0.5 mM each), RTCY primer (100 pmol), 30 min 50°C, 10 min 85°C.

**Table 2:** Summary of different experimental setups.

**Experiment**	**Animals**	**Antigen**	**Cells used**	**Isotype**	**Analysis**
#1	4 wt and 4 Tg	OVA	CD138^+^ cells	IgG	Length distribution analysis
#2	2 wt and 2 Tg	non-immunized	CD138^+^ cells	IgG	NGS
#3	6 wt and 6 Tg	OVA	CD138^+^ cells	IgG	NGS
#4	4 wt and 4 Tg	OVA	OVA specific CD19^+^ cells	IgG	NGS
#5	3 wt and 3 Tg	OVA	OVA specific CD19^+^ cells	IgM	NGS
#6	3 wt and 4 Tg	HEK-ABCC1	CD138^+^ cells	IgG	NGS
#7	4 wt and 4 Tg	3T3-ABCC1	CD138^+^ cells	IgG	NGS

*In the case of experiment 6 and 7 only a quarter of the spleen was used*.

PCR amplification of the antibody variable region was conducted in total volume of 25 μl, containing 4 μl cDNA, 1.25 U DreamTaq (Thermo Fisher Scientific), 10^*^DreamTaq buffer, 0.3 mM dNTP (each), 3.5 mM MgCl_2_, and 0.1 μM V_H_ specific primer (each) that anneal to framework region 1 (26). Following an initial denaturation step at 95°C for 5 min, PCR was conducted with 40 cycles of denaturation at 94°C for 50 s, annealing at 62°C for 50 s, and extension at 72°C for 50 s, with a final extension step at 72°C for 5 min. The IgG-specific reverse primers were fluorescently labeled with FAM [primers are listed in (26)]. This amplification results in apr. 430–545 bp length fragments, and an aliquot of 5 μl of each PCR amplicon was electrophoresed on 1.5% agarose gel for size check and 10 μl from each sample was sent for capillary gel electrophoresis (Biomi Ltd, Hungary). The returned data were analyzed with the Peak Scanner software (Applied Biosystems).

Using this tool, the diversity of the antibody repertoire can be characterized by two measures: the number of peaks of different V_H_ lengths and the distribution of these peaks. To describe this distribution, we used diversity indexes (Shannon index, Inverse Simpson index), adopted from ecology (20, 21). By definition, the values of these indexes are bigger if there are a greater number of different length peaks in the sample, and/or the distribution of sequence lengths is more even.

### cDNA Library Generation for Next Generation Sequencing

Total RNA was purified from CD138^+^ or OVA^+^CD19^+^ splenocytes of wt and Tg animals (Table 2) using RNeasy plus mini kit (Qiagen) according to the manufacturer's protocol. RNA concentration was measured on a Nanodrop ND-1000 spectrophotometer. The 5′ RACE approach was used for the generation of cDNA library to eliminate errors caused by multiplex primers. Oligo(dT)- or IgG-specific cDNA library was generated using SMARTScribe reverse transcriptase (Clontech) from 4 μl of total RNA, according to the manufacturer's protocol. Smart NNNNa was added for all samples, a 5′ adaptor carrying dU nucleotides (U) and a unique molecular identifier (UMI) consisting of 14 random “N” nucleotides (AAGCAGUGGTAUCAACGCAGAGUNNNNNUNNNNUNNNNNUCTTrGrGrGrG), for the template switch and cDNA synthesis reaction, which was carried out at 42°C for 60 min and terminated at 70°C for 15 min. cDNA synthesis products were treated with uracil-DNA glycosylase (New England BioLabs, 5 U per reaction) at 37°C for 30 min.

Two-stage PCR amplification was carried out to amplify the IgG-specific antibody variable region. For the first PCR, universal forward and IgG-specific reverse primers were used (primers are listed in Supplementary Table 1). The reaction was conducted in total volume of 20 μl, containing 1 μl cDNA, 0.5 U Phusion DNA polymerase (Thermo Fisher Scientific), 5^*^Phusion HF buffer, 0.4 mM dNTP (each), 0.1 μM primer (each). Following an initial denaturation step at 98°C for 30 s, PCR1 was conducted with 18 cycles of denaturation at 98°C for 15 s, annealing at 63°C for 20 s, and extension at 72°C for 20 s, with a final extension step at 72°C for 5 min. The product was gel electrophoresed in 1.5% agarose gel and the appropriate-sized product (550–650 bp) was gel excised and purified using NucleoSpin Gel and PCR Clean-up (Macherey-Nagel). For the second PCR 1 μl of purified PCR1 product was used per 50 μl. The same reaction conditions were used, except for the cycle number (25 instead of 18) and the primers (universal forward primer2 and IgG-specific reverse primer2 was used (Supplementary Table 1). The product was gel electrophoresed in 1.5% agarose gel and the appropriate sized product (500–600 bp) was gel excised and purified using NucleoSpin Gel and PCR Clean-up (Macherey-Nagel).

In the case of IgM-specific PCR, only the second PCR was performed, with 30 cycles, and an IgM-specific reverse primer.

Illumina adaptors were ligated using Ovation Ultralow Library System V2 (NuGEN) according to the manufacturer's protocol. The DNA concentration of the library was measured using Qubit and Real-time PCR, and libraries were submitted for a final quality control step on a Bioanalyzer 2100 (Agilent) prior to sequencing. Different barcodes were used for each sample, so that 30–35 samples could be analyzed in parallel.

### Sequencing and Bioinformatics

All samples were sequenced on the Illumina Miseq platform with 250 bp pair-end chemistry. The forward and reverse reads were error corrected (based on the UMI) using the MIGEC pipeline (27). The error corrected reverse reads with an average Phred base quality score of over 20 were selected and submitted to the IMGT/HighV-QUEST server (IMGT®, the international ImMunoGeneTics information system®). The output data was used to calculate several diversity measures (number of different clonotypes, the number of large clones, V gene usage) in Python and R, using the igraph package to identify clusters of related sequences (28). Statistical analyses were performed using GraphPad Prism 5 software. No correction was used for multiple comparisons.

### Flow Cytometry

For flow cytometry 3T3 and 3T3-ABCC1 or HEK and HEK-ABCC1 cells were used. The cells were counted, and the concentration was adjusted to 2E5 cell/well in FACS buffer (PBS, 1% FCS, Na-Azide). In each case 1:1,000 diluted sera (day 35 of immunization) of differently immunized wt and Tg mice were added to the cell suspensions on ice, for 25 min. The cell suspension was washed twice with 200 μl FACS buffer. Then eFluor 660 labeled goat anti-mouse antibody (Invitrogen) was added to each samples in 1:1,000 dilution for 25 min on ice. After washing twice with 200 μl FACS buffer, cells were resuspended in 100 μl FACS buffer. Samples were analyzed by flow cytometry using the high throughput plate function of the Cytoflex (BectonDickinson) instrument.

For positive control, MRPr1 (abcam) antibody was used in 1:50 dilution, which is specific for the inside loop of the ABCC1 receptor. Because of this, the cells were fixed (2% PFA, 10 min) and permeabilized (0.1% TritonX, 10 min) before labeling. The same protocol was used as described before, except Alexa® 488 labeled goat anti-rat antibody (abcam) was used as secunder antibody in 1:1,000 dilution.

## Results

### Increased Number of Splenocytes and Plasma Cells, and Higher Ab Titers in Tg Mice

**Figure 1:**
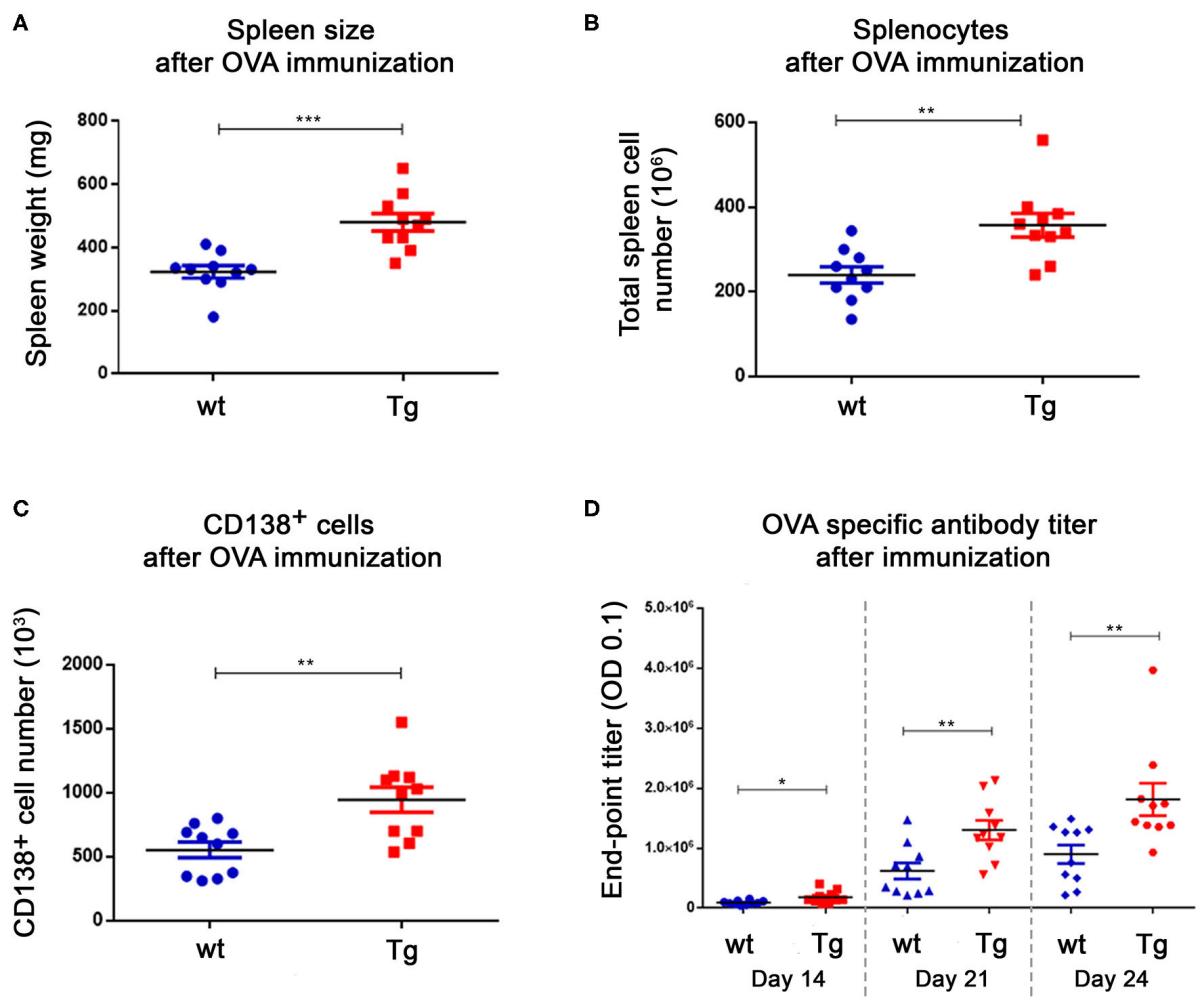
Immunization resulted in a great difference in the spleen size, the splenocyte number and the antigen specific antibody titer between the Tg and wt mice. After OVA immunization, animals were sacrificed on day 24. The weight of the spleens **(A)**, the number of splenocytes **(B)** and CD138^+^ cells **(C)** were measured. There was almost a 2-fold difference in favor of Tg animals in each case. Blood samples were collected on 3 different days during the immunization protocol and the antigen specific antibody titers were measured with ELISA (end-point titer: the dilution factor of the sera where the OD value is 0.1) **(D)**. The Tg animals had higher titers at all timepoints. Horizontal black lines and colored error bars represent the mean ± SEM of the data. Individual points correspond to specific animals. Differences between mean values were tested using unpaired *t*-tests. Statistically significant results are marked with asterisks (*p < 0.05, **p < 0.01, ***p < 0.001).

The ovalbumin immunized animals (10 wt and 10 Tg) were sacrificed on day 24 (Table 1). We measured the weight of the spleen and counted the number of splenocytes by Bürker chamber. Tg mice had larger spleens, and contained significantly more splenocytes compared to the wt animals (Figures 1A,B). After labeling the CD138^+^ cells, we sorted out nearly twice as many plasma cells from the Tg spleens (Figure 1C). Two-fold difference was detected in the case of the antigen specific antibody titers as well (Figure 1D). These results are in line with our previously reported observations (16).

### Length Distribution Analysis of the Heavy Chain Variable Regions Indicates Increased Diversity of B-Cell Response in Tg Mice

**Figure 2:**
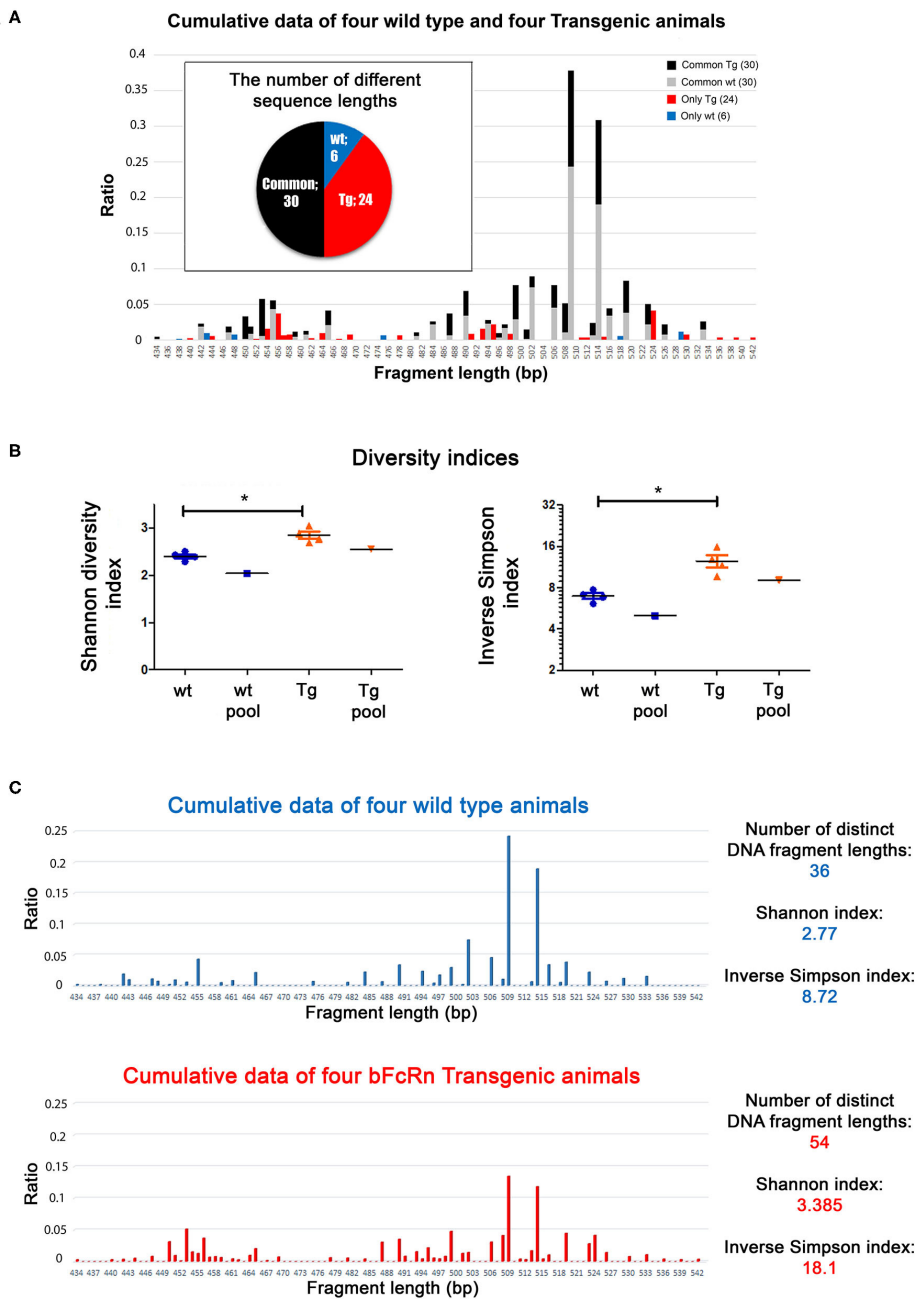
Length distribution analysis of the variable regions of the Tg and wt mice. The animals were immunized with OVA and were sacrificed on day 24. **(A)** Data from 4 wt and 4 Tg animals were summarized and illustrated in one graph. The Tg animals contained sequences with more distinct lengths (pie chart: 24 unique + 30 common = 54 Tg altogether vs. 6 unique + 30) common = 36 wt altogether (common: it was found in the wt and Tg samples as well) and their sequence length distribution was more even (bar chart). Sequence lengths unique to either wt or Tg mice are illustrated in blue and red, respectively. **(B)** Diversity indices (Shannon, Inverse Simpson) for wt and Tg samples. Horizontal black lines and colored error bars represent the mean ± SEM of the data. Individual points correspond to specific animals. Pooled columns represent results obtained when pooling samples at cDNA level. Differences between mean values were tested using Mann-Whitney test. Statistically significant results are marked with asterisks (*p < 0.05). **(C)** Length distribution analysis of the variable regions of Tg and wt mice, where the data from 4 wt and 4 Tg animals are illustrated in two individual graphs.

We performed a length distribution analysis using CD138^+^ cells from 4 wt and 4 Tg animals after OVA immunization. Tg animals produced 1.5 times more distinct length groups of IgG sequences (54 vs. 36 in the pooled data) and displayed 4 times as many unique peaks (24 vs. 6), compared to the wild type animals (Figure 2A). The diversity indices show that Tg animals had a more diverse length distribution, compared to wt mice (Figure 2B), even when we pooled either the spectratyping data derived from the animals after the analysis (Figure 2C), or the cDNAs before the reaction (Supplementary Figure 1A). These data clearly show that Tg animals had a more diverse immune repertoire after OVA immunization.

### The Strategy of the NGS Analysis, Bioinformatics Pipeline

Different experiment strategies were set up to analyze the diversity of the B-cell repertoire of Tg and wt mice by NGS. We used different antigens, immunization schedules and analyzed different cells and Ig isotypes to perform a deep investigation of the repertoires (Table 2). A unique molecular identifier (UMI) was added to all sequences to allow for an UMI-based error correction pipeline and to eliminate PCR bias, using the MIGEC tool (27). The error corrected sequences were uploaded to the IMGT/HighV-QUEST server and only sequences deemed “productive” have been selected for further analysis steps. We used different measures to investigate the diversity of the samples. There is no clear agreement in literature on which B-cell clone definition is the most suitable and best available, so we used two approaches in our analyses. First, we determined the number of different CDR3 sequences at the amino acid (AA) level (“CDR3”). Second, beside the CDR3 region, the origins of the V and J gene segments (obtained from the IMGT analysis) were considered as well (“CDR3VJ”). In addition, we calculated the number of dominant clones in the samples. We considered a clone dominant if the ratio of its supporting sequences (defined at the nucleotide level) exceeded 0.5% of all sequences in the given sample (29). If a sample contains a greater number of dominant clones, the diversity is smaller because the distribution of sequences is less even. To assess if there was a difference in the diversity of activated naïve B cells between the samples, two further analyses were performed. First, we determined the number of distinct sequences in the samples that did not contain any deviation from the germline sequence in the V and J genes (VJ100), and can therefore be assumed to be derived from a naïve B cell. Second, we carried out a network analysis with individual sequences as nodes, where sequences that differed only in one amino acid in their CDR3 region were linked (edges). Due to the high similarity of linked sequences, clusters of the graph represent a group of sequences likely originating from a single common naïve B cell. Thus, the number of clusters can be interpreted as the number of different activated naïve B cells in the given animal.

In conclusion, a sample with a highly diverse immune repertoire should contain many different sequence clones (regardless of the exact definition) and a large number of clusters in its graph (thus many different naïve B cells), and single clones or clusters should not be supported by an overly large ratio of the sequenced reads.

### Increased Diversity of CD138^+^ Plasma Cells Before and After OVA Immunization in Tg Mice

**Figure 3:**
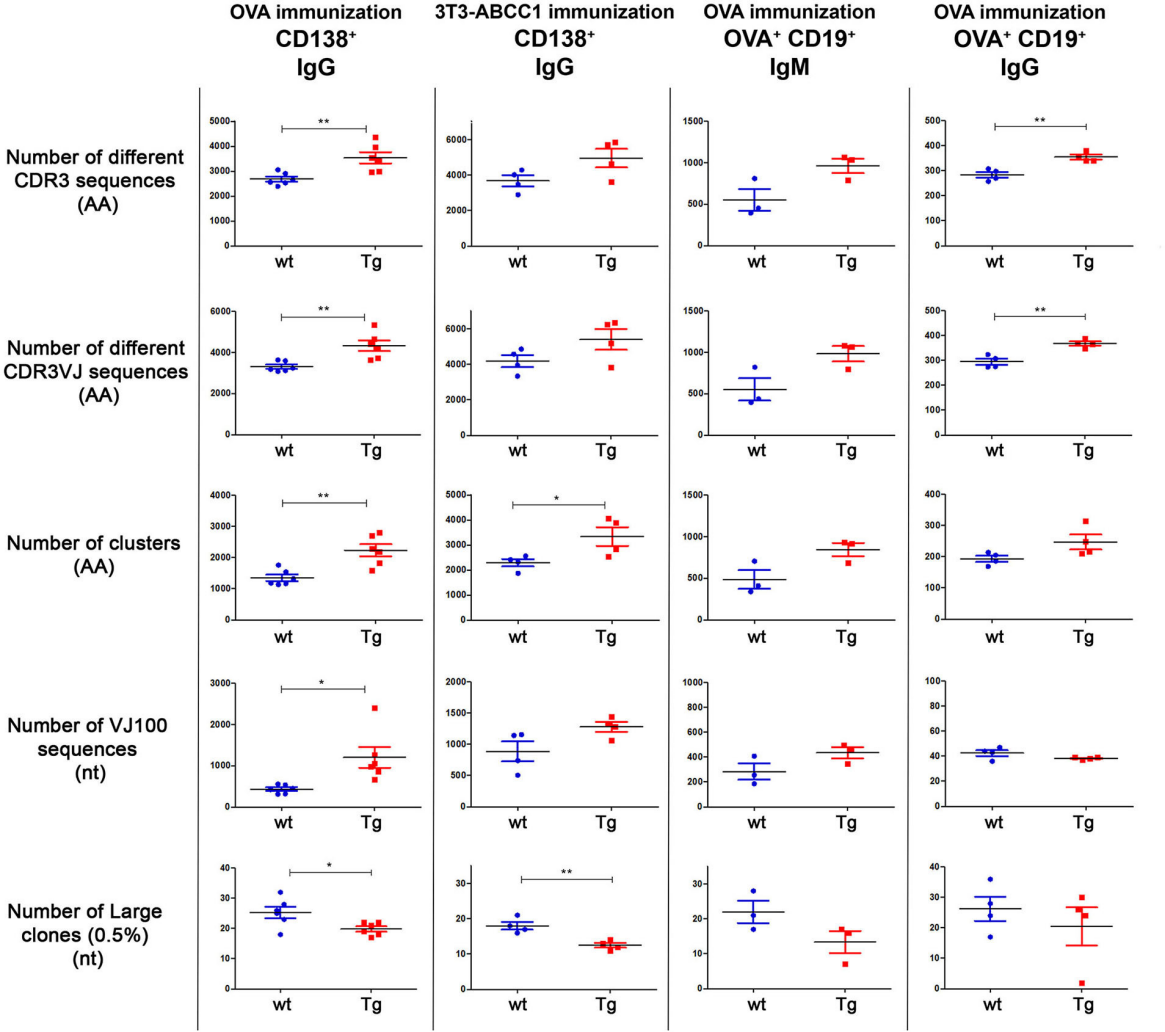
Summary of diversity measures obtained from NGS analysis. The animals were immunized with OVA or cellular antigens and were sacrificed on day 24 or day 66 (see Table 1). Different cell types (CD138^+^ or CD19^+^OVA^+^) were sorted and the Ig repertoires were analyzed by NGS. Results for different diversity measures are plotted in each row, with various immunization protocol, antigen, cell type, and Ig isotype combinations represented in each column. Number of different CDR3 sequences (AA): Sequences with different CDR3 region at the amino acid level. Number of different CDR3VJ sequences (AA): Sequences with different CDR3 region at the amino acid level and with different V and J gene usage. Number of clusters (AA): Sequences that differ only in one amino acid in their CDR3 region were grouped into one cluster. Number of VJ100 sequences (nt): Different nucleotide sequences in the samples that did not contain any deviation from the germline sequence in the V and J genes. Number of large clones (0.5%) (nt): We considered a clone large if the proportion of its nucleotide sequences exceeded 0.5% of the repertoire. When a sample contains more dominant clones the diversity is lower as the distribution is less even. Horizontal black lines and colored error bars represent the mean ± SEM of the data. Individual points correspond to specific animals. Differences between mean values were tested using unpaired *t*-tests. Statistically significant results are marked with asterisks (*p < 0.05, **p < 0.01).

**Figure 4:**
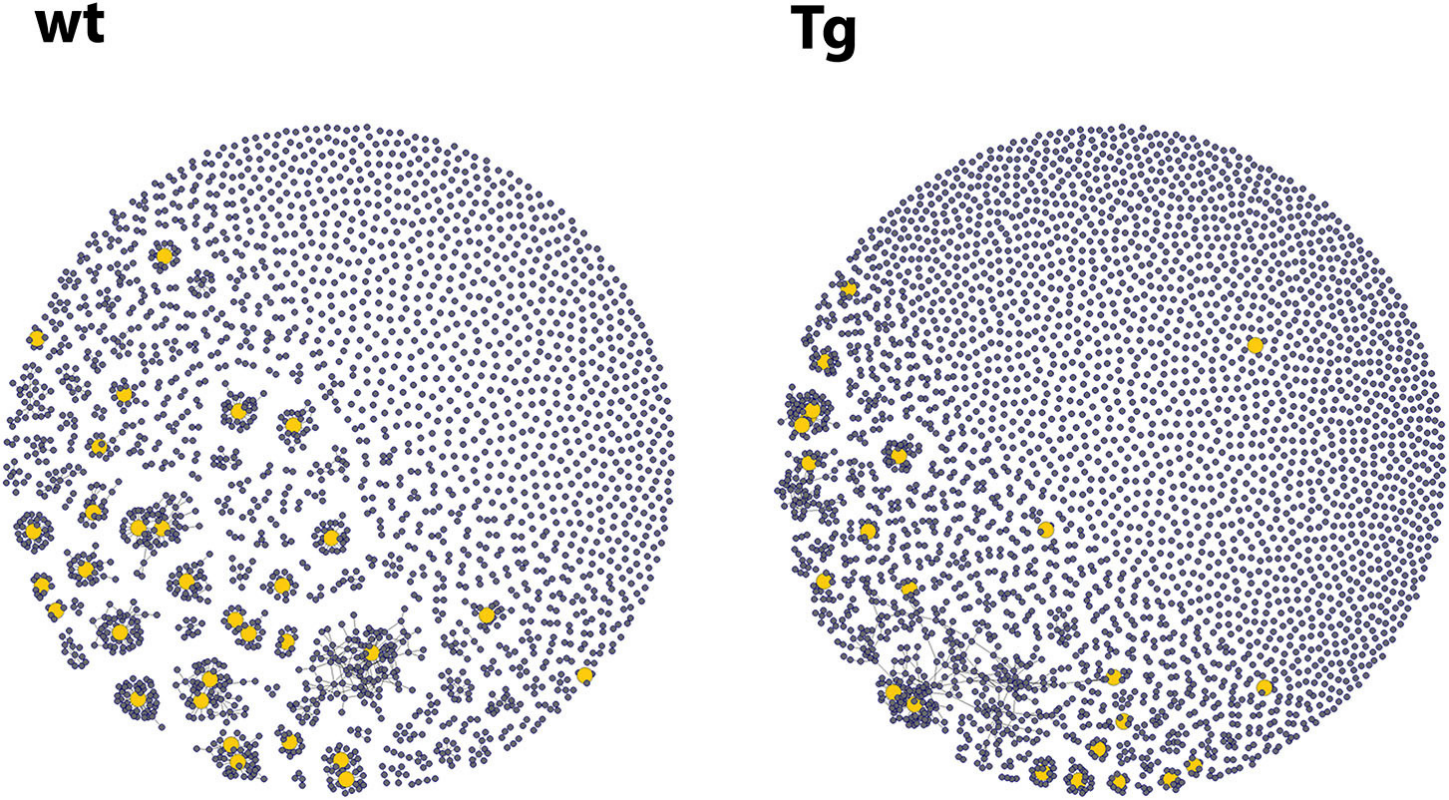
Representative graphs from the network analysis of the repertoires of CD138^+^ plasma cells after OVA immunization. Each vertex of the graph represents a single sequence with a link (edges) to those sequences that did not differ from it in more than one nucleotide. Vertex size corresponds to the number of identical sequences found. The yellow vertices represent highly expanded clones (each harboring at least 0.5% of the sequences). The Tg animals contained more clusters (mean: 2,229 vs. 1,347), which suggests that more naïve B cell clones were activated in them upon immunization. The graphs were prepared using the igraph R package.

In our first experiment, 6 wt and 6 Tg animals were immunized with OVA using the same protocol as for spectratyping (Table 2), then CD138^+^ cells were sorted (Supplementary Figure 2A) and the CDR3 (VJ) genomic region was analyzed by NGS. We observed that Tg animals produced a significantly larger number (~1.3 times more than wt mice) different CDR3 and CDR3VJ clones (Figure 3, Supplementary Table 2). They contained significantly fewer large clones as well, which also suggests, that they have a more diverse immune repertoire. After this assessment, we performed a network analysis (Figure 4) and determined the number of unmutated V and J sequences (VJ100, no deviation from the germline sequence). The results indicate that Tg mice contained more than twice as many different VJ100 sequences and harbored a 1.6 times more clusters compared to the wild type animals, suggesting that the original number of responding naïve B cell clones was also higher in these animals (Figure 3). We analyzed the V and J gene usage (data not shown) and the number of mutations in the V gene as well (Supplementary Figure 3), but we did not find significant differences between the animals.

We also analyzed the CD138^+^ cells of non-immunized animals, and could observe little difference between the animals (Supplementary Figure 4). Remarkably, the number of distinct CDR3 or CDR3VJ sequences was about 4- or 5-fold smaller in the non-immunized compared with immunized animals, indicating that the majority of the repertoire after immunization arose in response to the challenge. Although sample size was small, these observations suggest that the difference in diversity of CD138^+^ B-cell repertoire in the previous experiment was probably due to the different efficiency of immunization. In addition, if we subtract the number of CDR3 or CDR3VJ clones we got from the non-immunized animals from those of immunized ones, the difference between the animals are even more pronounced, i.e., Tg mice had ~1.4 times more new CDR3 or CDR3VJ clones after immunization that wt ones.

It is well-known that the CDR3 length distributions tend to be normally distributed. In the present study, the length distribution of the wt and Tg mice were analyzed through test of normality after immunization (Supplementary Figures 5A,B). We found that the CDR3 length distribution of Tg animals was very similar to that of the wt animals, and both conformed to normality.

### Increased Diversity of OVA Specific CD19^+^ Cells After OVA Immunization in Tg Mice

To investigate the antigen specific B-cell repertoires of the animals, we sorted the CD19^+^ OVA specific B cells from spleens of OVA immunized Tg and wt mice (Supplementary Figure 2B) and generated IgG- and IgM-specific cDNA libraries from these samples for NGS analyses.

At IgG level, we detected a 1.25 times increase in the number of different clones, 0.78 times reduction in the number of large clones, 1.28 times increase in the number of different clusters, and essentially no difference in the number of VJ100 sequences in the Tg mice as compared to their wt controls (Figure 3, Supplementary Figure 6). Of these, the changes in the number of distinct clones were statistically significant.

At IgM level, the differences between the two groups were even more pronounced. The Tg animals had nearly twice as many different clones (1.75 times), and half as many large clones (0.6 times) than wt mice. Tg mice also had 1.53 times more distinct VJ100 sequences and 1.74 times more clusters as well (Figure 3, Supplementary Figure 6). However, due to the small number of animals, none of these results were statistically significant.

We also analyzed the number of mutations in the V genes, and found no difference between the two groups. As could be expected, IgM sequences contained significantly fewer mutations than IgG sequences in both groups of mice (Supplementary Figure 3B). The smaller diversification of IgM was also apparent in the weaker connectivity of the networks based on sequence similarity (Supplementary Figures 6B,C).

### Increased Diversity of CD138^+^ Cells After Transfected Cell Immunization in Tg Mice

Currently, there are many mAb discovery protocols for cell membrane proteins. Thus, as a next step, we used transfected cells and a more challenging target (ABCC1 plasma membrane protein) for immunization and analyzed the diversity of the immune response.

After immunization with 3T3-ABCC1 cells, the Tg animals appeared to produce a larger number of distinct CDR3 and CDR3VJ clones (1.35 times and 1.29 times), they contained fewer large clones (0,69 times), and had more VJ100 sequences (1.44 times) and clusters (1.46 times) as well (Figure 3, Supplementary Figure 6). These results are very similar to what we observed in the case of ovalbumin immunization, however, the observed differences were only significant for the number of clusters and large clones.

The immunological advantage of Tg animals could be verified in the case of HEK-ABCC1 immunization as well. They also contained more distinct clones (CDR3 1.23 times), fewer large clones (0.75 times) and more clusters (1.16 times), but there was no difference in the number of VJ100 sequences (0.96 times) (Supplementary Figure 7). Of these results, only the comparison of the numbers of differently defined clones (CDR3 and CDR3VJ) were statistically significant.

### Tg Mice Developed Enhanced ABCC1 Specific Antibody Response

The ABCC1 plasma membrane protein is a highly conserved protein among mammals, there is only a subtle difference between the human and murine variants. Due to this similarity, it is very difficult to induce an effective immune response against this human target in mouse. To validate the efficiency of immunization in the Tg animals in such a challenging test case, we analyzed the presence of ABCC1 specific antibodies in the sera of immunized mice by flow cytometry. We tested the sera of the HEK-ABCC1 immunized mice using ABCC1 transfected 3T3 cells (Supplementary Figure 2C) and their parental non-transfected cells, and the sera of 3T3-ABCC1 immunized mice with HEK and HEK-ABCC1 cells. The results showed that both immunizations produced antibodies on day 35 immunization that recognized the other cell type (shared epitopes), and sera from Tg animals showed a greater shift on receptor expressing cells compared to control cells (especially in the case of HEK immunizations) suggesting that they generated (more) ABCC1 specific antibodies (Supplementary Figure 8). In the case of the 3T3 immunizations the signals and the differences between the two groups were less pronounced.

## Discussion

In the last 30 years monoclonal antibodies have had an emerging role in the fields of research, diagnostics and therapeutics, as well. There are many challenging targets that would be worth investigating (for example highly conserved and hidden membrane proteins), but their immunogenicity is very low, and therefore there is no antibody against them. One of the promising approaches to improve currently available antibody producing methods is based on bFcRn transgenic mice (14, 30). Multiple studies have demonstrated that high-level expression of bFcRn can enhance the immune response of these animals, resulting in higher antigen specific antibody titers, more antigen specific B and T helper cells, and more and bigger germinal centers in the spleen following immunization (16). In addition, these animals have been used successfully to generate antibodies against weakly immunogenic targets (13), and an epitope mapping study demonstrated a high diversity of the produced antibodies (14).

To better understand the biological mechanisms of the superior immunization efficiency in bFcRn transgenic mice, we used different immunization strategies and methods to analyze the antibody repertoire of these animals. Our results were in agreement with previous observations: Tg animals had higher antigen specific antibody titers, bigger spleens, more splenocytes, and almost twice as many CD138^+^ plasma cells as the wild type mice. The main focus of our research was to determine whether the higher number of plasma cells in Tg animals also correlates with a higher sequence diversity or is merely the effect of high abundance of the same type of cells. To address this question CD138^+^ cells were sorted out and the diversity of the antibodies was analyzed with a length distribution analysis of the variable regions and next generation sequencing (NGS).

First, we analyzed the length distribution of IgG sequences after OVA immunization. In this analysis, the size of a given fragment is affected by several factors: the binding site of the forward primer, the length of the V gene, and the length of the CDR3 region. This method is less sensitive and has a lower depth compared to modern techniques like NGS, but is able to provide a comprehensive picture of the condition of the animals and has been used for rapid and inexpensive analysis of the antibody repertoire (31–33). The diversity of a single sample was measured by calculating different parameters of the length distribution mainly used in ecological studies (number of peaks, Shannon diversity index and inverse Simpson index) (20, 21). By analyzing 4 wt and 4 Tg mice together we were able to show, that under the same conditions the transgenic animals contained more fragments of different lengths and the distribution of these fragments was more even, indicating a more diverse antibody response in Tg animals.

To confirm and better characterize the advantage of Tg animals, we also performed immunoglobulin repertoire analysis with NGS (Rep-seq), which yields a higher resolution survey of the antibody repertoire (19, 24, 34, 35). Rep-seq is a powerful tool for obtaining vast amounts of information about the antibody repertoire and it's utilization is emerging in various fields, for example analyzing the effects of vaccinations (34, 36), aging (37), hematological malignancies (38), autoimmune diseases (39) or simply the normal repertoire (40), either in humans or in mice (41).

As it is the expression level of different antibodies that is relevant for practical purposes, we used mRNA as a starting material for sequencing (19). The main purpose of the study was not to analyze the complete repertoire of mice, which would have required a much deeper sequencing (40), but to compare the animals at a lower depth under a wide variety of circumstances. A number of different metrics were used to evaluate the results and to compare the repertoires.

Following immunization with ovalbumin, analysis of the repertoires revealed that the CD138^+^ plasma cells of Tg mice carried a more diverse repertoire of antibody sequences. Comparing this data to the results of unimmunized animals showed that the difference in the number of clonotypes generated upon immunization is even more pronounced. This difference may reflect enhanced somatic hypermutation (SHM) and/or a larger number of activated naïve B-cell clones. To determine this, we used network analysis and determined the number of unmutated VJ100 sequences. Both approaches suggested that the difference was not merely a result of enhanced SHM, but a higher number of different naïve B cells had apparently been activated. This result is in agreement with our previous studies where we found that the Tg animals had more germinal centers in the spleen after immunization (16). Further analyses revealed that both groups had normally distributed antibody repertoires, and there was no significant difference in the use and mutation frequency of V and J genes. The comparable mutation frequencies may suggest that the FcRn is not involved in affinity maturation, but further experiments involving longer immunization protocols and different methods (affinity assays) will be needed to confirm this conclusion.

To assess the diversity of antigen specific B cells, CD19 and OVA double positive cells were sorted out from the animals (42) after a similar immunization protocol and the diversity of these B cells was examined at IgG and IgM level as well. Using the IgG library, we obtained similar results to that of the CD138^+^ plasma cells, and an even greater difference at the IgM level could be demonstrated between the Tg and wt animals. The latter is in line with the results of our previous epitope mapping study (14), but we were able to demonstrate the higher diversity of the antibodies at IgG level for the first time. This might be the result of examining the diversity at the level of nucleotides, or focusing solely on the CD19+ B cells instead of the whole antibody repertoire. It is also likely that due to a shorter immunization protocol there was not enough time for epitope exclusion, which tends to select antibodies that can recognize only the immunodominant epitope(s) (14, 43). As a consequence, we were able to detect less abundant antibody sequences, as well, that target non-dominant epitopes. The CDR3 lengths (IgM and IgG) of the sequences are also in line with previously published data (44, 45).

We also used immunization protocols with cellular antigens (3T3-ABCC1 and HEK-ABCC1). The principal difference between the two antigens was that 3T3 is a mouse cell line and therefore has smaller immunogenicity to activate the normal murine immune system. Using these cells, we also obtained more diverse B-cell responses in Tg mice, although the differences were smaller, suggesting that the longer immunization protocol may have focused the immune repertoire., when we were immunizing with HEK-ABCC1, Tg mice produced substantially more ABCC1 specific antibodies compared to wt animals, demonstrating the utility of this approach in the challenging setting of cellular immunization with highly conserved proteins. In the meanwhile, we have submitted a manuscript that describes a successful mAb discovery process using transfected cells and the same immunization protocol (this mAb recognizes an extracellular epitope of the human ABCC6 (Kozak et al. submitted). This data also confirms the improved antibody production of these bFcRn Tg mice using transfected cell immunization protocol. Since we used CFA and IFA as adjuvants, we assume that some of the immune response was elicited by the CFA/IFA themselves and are not antigen specific.

The greater diversity of the antibody repertoire and the activation of more naïve B cells after immunization in bFcRn Tg animals is consistent with our earlier hypothesis on the B-cell responses of these animals (46). We conjectured that the overexpression of FcRn sequesters more antigen specific IgGs in the circulation, which, during a secondary immune response, allows antigen presenting cells to take up more immune complexes and to present more epitopes, derived from the antigen, on their surface. As a result, more antigen specific naïve B and T helper cells will be activated, and the diversity of the humoral immune response will be greater. Based on this, the FcRn along with its many other roles, has an important function in the generation of highly diverse immune repertoire upon immunization.

Finally, we note that our analyses were limited to nucleotide or amino acid sequence information, thus we had no information on what epitopes were recognized by the antibodies detected at the CDR3 sequence level. Further studies using, for example epitope binning, will be needed to characterize epitope targeting, and it would be very interesting to analyze the T helper cell repertoire as well. T helper cells in the spleen have a key role in B-cell activation, and a more diverse B-cell repertoire might hint at a greater T helper cell diversity, as well.

In conclusion, our findings provide further support that bFcRn Tg animals develop a more potent and more diverse B-cell response upon immunization. Ovalbumin is an immunogenic antigen, therefore it is easy to produce antibodies against it in normal animals as well, but the greater diversity of the repertoire suggests that Tg animals produce a broader set of antibodies against its epitopes. This difference is likely to make these animals an improved tool for mAb discovery. Indeed, with the use of the bFcRn Tg mice, we have already produced a number of specific antibodies that are capable of recognizing weakly immunogenic antigens (13, 17) and also have functional effects (e.g., receptor-inhibition) (18). The use of these animals may continue to help to produce antibodies with sufficient specificity and quality against other difficult targets.

## Data Availability Statement

The datasets generated for this study can be found in the SRA (Sequence read archive) server. SRA accession: PRJNA599097. Release date: 2020-05-25. All the sequences are accessible with the following link: https://www.ncbi.nlm.nih.gov/sra/PRJNA599097.

## Ethics Statement

The animal study was reviewed and approved by Food Chain Safety and Animal Health Directorate of the Government Office of Pest County, Hungary (permissions PEI/001/2196-2/2013).

## Author Contributions

BS contributed in most of the experiments (immunization, cell sorting, cDNA library production, next generation sequencing, bioinformatic evaluation). AM contributed in the analysis of the non-immunized animals. PJ contributed in the sorting of the CD138+ plasma cells. OP and IC contributed in the bioinformatic evaluations. VM contributed in the evaluation of the spectratyping data (ecological indexes). IK conceived and supervised the project and wrote the manuscript. All authors contributed to the article and approved the submitted version.

## Conflict of Interest

BS, PJ, and IK are employees of ImmunoGenes using patented, genetically modified animal, that overexpress FcRn for the production of polyclonal and monoclonal antibodies. The remaining authors declare that the research was conducted in the absence of any commercial or financial relationships that could be construed as a potential conflict of interest.

## References

[1] Brambell FWR Hemmings WA Morris IG. A theoretical model of gammaglobulin catabolism. Nature. (1964) 203:1352–5. 10.1038/2031352a0 14207307

[2] Brambell FW. The transmission of immune globulins from the mother to the foetal and newborn young. Proc Nutr Soc. (1969) 28:35–41. 10.1079/PNS19690007 4182340

[3] Jones EA Waldmann TA. The mechanism of intestinal uptake and transcellular transport of IgG in the neonatal rat. J Clin Invest. (1972) 51:2916–27. 10.1172/JCI107116 5080417 PMC292442

[4] Rodewald R Kraehenbuhl JP. Receptor-mediated transport of IgG. J Cell Biol. (1984) 99:159s−64s. 10.1083/jcb.99.1.159s 6235233 PMC2275593

[5] Simister NE Rees AR. Isolation and characterization of an Fc receptor from neonatal rat small intestine. Eur J Immunol. (1985) 15:733–8. 10.1002/eji.1830150718 2988974

[6] Simister NE Mostov KE. An Fc receptor structurally related to MHC class I antigens. Nature. (1989) 337:184–7. 10.1038/337184a0 2911353

[7] Ward ES Ober RJ. Chapter 4: multitasking by exploitation of intracellular transport functions the many faces of FcRn. Adv Immunol. (2009) 103:77–115. 10.1016/S0065-2776(09)03004-1 19755184 PMC4485553

[8] Roopenian DC Akilesh S. FcRn: the neonatal Fc receptor comes of age. Nat Rev Immunol. (2007) 7:715–25. 10.1038/nri2155 17703228

[9] Baker K Rath T Pyzik M Blumberg RS The role of FcRn in antigen presentation. Front Immunol. (2014) 5:408 10.3389/fimmu.2014.00408 25221553 PMC4145246

[10] Bender B Bodrogi L Mayer B Schneider Z Zhao Y Hammarstrom L. Position independent and copy-number-related expression of the bovine neonatal Fc receptor alpha-chain in transgenic mice carrying a 102 kb BAC genomic fragment. Transgenic Res. (2007) 16:613–27. 10.1007/s11248-007-9108-9 17594529

[11] Catunda Lemos AP Cervenak J Bender B Hoffmann OI Baranyi M Kerekes A. Characterization of the rabbit neonatal Fc receptor (FcRn) and analyzing the immunophenotype of the transgenic rabbits that overexpresses FcRn. PLoS ONE. (2012) 7:e28869. 10.1371/journal.pone.0028869 22247762 PMC3256154

[12] Cervenak J Bender B Schneider Z Magna M Carstea BV Liliom K. Neonatal FcR overexpression boosts humoral immune response in transgenic mice. J Immunol. (2011) 186:959–68. 10.4049/jimmunol.1000353 21148035

[13] Vegh A Cervenak J Jankovics I Kacskovics I. FcRn overexpression in mice results in potent humoral response against weakly immunogenic antigen. mAbs. (2011) 3:173–80. 10.4161/mabs.3.2.14462 21239891 PMC3092618

[14] Vegh A Farkas A Kovesdi D Papp K Cervenak J Schneider Z. FcRn overexpression in transgenic mice results in augmented APC activity and robust immune response with increased diversity of induced antibodies. PLoS ONE. (2012) 7:e36286. 10.1371/journal.pone.0036286 22558422 PMC3340356

[15] Baranyi M Cervenak J Bender B Kacskovics I. Transgenic rabbits that overexpress the neonatal Fc receptor (FcRn) generate higher quantities and improved qualities of anti-thymocyte globulin (ATG). PLoS ONE. (2013) 8:e76839. 10.1371/journal.pone.0076839 24194847 PMC3806768

[16] Schneider Z Jani PK Szikora B Vegh A Kovesdi D Ilias A. Overexpression of bovine FcRn in mice enhances T-dependent immune responses by amplifying T helper cell frequency and germinal center enlargement in the spleen. Front Immunol. (2015) 6:357. 10.3389/fimmu.2015.00357 26257730 PMC4507463

[17] Dudok B Barna L Ledri M Szabo SI Szabadits E Pinter B. Cell-specific STORM super-resolution imaging reveals nanoscale organization of cannabinoid signaling. Nat Neurosci. (2015) 18:75–86. 10.1038/nn.3892 25485758 PMC4281300

[18] Vaeth M Yang J Yamashita M Zee I Eckstein M Knosp C. ORAI2 modulates store-operated calcium entry and T cell-mediated immunity. Nat Commun. (2017) 8:14714. 10.1038/ncomms14714 28294127 PMC5355949

[19] Georgiou G Ippolito GC Beausang J Busse CE Wardemann H Quake SR. The promise and challenge of high-throughput sequencing of the antibody repertoire. Nat Biotechnol. (2014) 32:158–68. 10.1038/nbt.2782 24441474 PMC4113560

[20] MagurranAE Measuring Biological Diversity. Oxford: Wiley-Blackwell (2004).

[21] Six A Mariotti-Ferrandiz ME Chaara W Magadan S Pham HP Lefranc MP. The past, present, and future of immune repertoire biology - the rise of next-generation repertoire analysis. Front Immunol. (2013) 4:413. 10.3389/fimmu.2013.00413 24348479 PMC3841818

[22] Pannetier C Levraud JP Lim A Even J Kourilsky P The Immunoscope approach for the analysis of T-cell repertoires. In: Oksenberg J, editor. The Human Antigen T-cell Receptor: Selected Protocols and Applications. Austin, TX: R.G. Landes Company (1996). p. 162.

[23] Weinstein JA Jiang N White RA 3rd Fisher DS Quake SR. High-throughput sequencing of the zebrafish antibody repertoire. Science. (2009) 324:807–10. 10.1126/science.1170020 19423829 PMC3086368

[24] Greiff V Menzel U Haessler U Cook SC Friedensohn S Khan TA. Quantitative assessment of the robustness of next-generation sequencing of antibody variable gene repertoires from immunized mice. BMC Immunol. (2014) 15:40. 10.1186/s12865-014-0040-5 25318652 PMC4233042

[25] Yaari G Kleinstein SH. Practical guidelines for B-cell receptor repertoire sequencing analysis. Genome Med. (2015) 7:121. 10.1186/s13073-015-0243-2 26589402 PMC4654805

[26] Rohatgi S Ganju P Sehgal D. Systematic design and testing of nested (RT-)PCR primers for specific amplification of mouse rearranged/expressed immunoglobulin variable region genes from small number of B cells. J Immunol Methods. (2008) 339:205–19. 10.1016/j.jim.2008.09.017 18926828

[27] Shugay M Britanova OV Merzlyak EM Turchaninova MA Mamedov IZ Tuganbaev TR. Towards error-free profiling of immune repertoires. Nat Methods. (2014) 11:653–5. 10.1038/nmeth.2960 24793455

[28] Csardi G Nepusz T The igraph software package for complex network research. InterJ Complex Syst. (2006) 1965. Available online at: https://cran.r-project.org/web/packages/igraph/citation.html.

[29] Tak PP Doorenspleet ME de Hair MJH Klarenbeek PL van Beers-Tas MH van Kampen AHC. Dominant B cell receptor clones in peripheral blood predict onset of arthritis in individuals at risk for rheumatoid arthritis. Ann Rheum Dis. (2017) 76:1924–30. 10.1136/annrheumdis-2017-211351 28790026 PMC5705849

[30] Hutchings CJ Koglin M Marshall FH. Therapeutic antibodies directed at G protein-coupled receptors. MAbs. (2010) 2:594–606. 10.4161/mabs.2.6.13420 20864805 PMC3011214

[31] Miqueu P Guillet M Degaugue N Doré JC Soulillou JP Brouard S. Statistical analysis of CDR3 length distributions for the assessment of T and B cell repertoire biases. Mol Immunol. (2007) 44:1057–64. 10.1016/j.molimm.2006.06.026 16930714

[32] Ademokun A Wu YC Martin V Mitra R Sack U Baxendale H. Vaccination-induced changes in human B-cell repertoire and pneumococcal IgM and IgA antibody at different ages. Aging Cell. (2011) 10:922–30. 10.1111/j.1474-9726.2011.00732.x 21726404 PMC3264704

[33] Foreman AL Lemercier B Lim A Kourlisky P Kenny T Gershwin ME. VH gene usage and CDR3 analysis of B cell receptor in the peripheral blood of patients with PBC. Autoimmunity. (2008) 41:1:80–86. 10.1080/08916930701619656 18176868

[34] Galson JD Trück J Fowler A Clutterbuck EA Münz M Cerundolo V. Kelly analysis of B cell repertoire dynamics following hepatitis B vaccination in humans, and enrichment of vaccine-specific antibody sequences. EBioMedicine. (2015) 2:2070–9. 10.1016/j.ebiom.2015.11.034 26844287 PMC4703725

[35] Simchoni N Cunningham-Rundles C. TLR7 and TLR9 responsive human B cells share phenotypic and genetic characteristics. J Immunol. (2015) 194:3035–44. 10.4049/jimmunol.1402690 25740945 PMC4369401

[36] Li R Fu F Feng L Liu P. Next-generation sequencing and single-cell RT-PCR reveal a distinct variable gene usage of porcine antibody repertoire following PEDV vaccination. Sci China Life Sci. (2019) 17:1–11. 10.1007/s11427-019-9576-2 31321668 PMC7088813

[37] Tabibian-Keissar H Hazanov L Schiby G Rosenthal N Rakovsky A Michaeli M. Aging affects B-cell antigen receptor repertoire diversity in primary and secondary lymphoid tissues. Eur J Immunol. (2016) 46:480–92. 10.1002/eji.201545586 26614343

[38] Müller DJ Wirths S Fuchs AR Märklin M Heitmann JS Sturm M. Loss of NFAT2 expression results in the acceleration of clonal evolution in chronic lymphocytic leukemia. J Leukoc Biol. (2019) 105:531–8. 10.1002/JLB.2AB0218-076RR 30556925

[39] Yan Q Wang L Lai L Liu S Chen H Zhang J. Next generation sequencing reveals novel alterations in B-cell heavy chain receptor repertoires associated with acute-on-chronic liver failure. Int J Mol Med. (2019) 43:243–55. 10.3892/ijmm.2018.3946 30365073 PMC6257861

[40] Briney B Inderbitzin A Joyce C Burton DR. Commonality despite exceptional diversity in the baseline human antibody repertoire. Nature. (2019) 566:393–7. 10.1038/s41586-019-0879-y 30664748 PMC6411386

[41] Chaaya N Shahsavarian MA Maffucci I Friboulet A Offmann B Léger JB. Genetic background and immunological status influence B cell repertoire diversity in mice. Sci Rep. (2019) 9:14261. 10.1038/s41598-019-50714-y 31582818 PMC6776527

[42] von Boehmer L Liu C Ackerman S Gitlin AD Wang Q Gazumyan A. Sequencing and cloning of antigen-specific antibodies from mouse memory B cells. Nat Protoc. (2016) 11:1908–23. 10.1038/nprot.2016.102 27658009

[43] Agarwal A Sarkar S Nazabal C Balasundaram G Rao KV. B cell responses to a peptide epitope. I. The cellular basis for restricted recognition. J Immunol. (1996) 157:2779–88. 8816380

[44] Rettig TA Ward C Bye BA Pecaut MJ Chapes SK. Characterization of the naive murine antibody repertoire using unamplified high-throughput sequencing. PLoS ONE. (2018) 13:e0190982. 10.1371/journal.pone.0190982 29320559 PMC5761896

[45] Shi B Ma L He X Wang X Wang P Zhou L. Comparative analysis of human and mouse immunoglobulin variable heavy regions from IMGT/LIGM-DB with IMGT/HighV-QUEST. Theor Biol Med Model. (2014) 11:30 10.1186/1742-4682-11-30 24992938 PMC4085081

[46] Cervenak J Kurrle R Kacskovics I. Accelerating antibody discovery using transgenic animals overexpressing the neonatal Fc receptor as a result of augmented humoral immunity. Immunol Rev. (2015) 268:269–87. 10.1111/imr.12364 26497527

