# Supplementary material for: FcRn Overexpression Expands Diversity of the Humoral Immune Response in bFcRn Transgenic Mice
Source: Front Immunol. 2020 Aug 21;11:1887. doi: 10.3389/fimmu.2020.01887 (PMC7472951; doi:10.3389/fimmu.2020.01887)
Supplement: Supplementary file 1 [file Data_Sheet_1.PDF]

Supplementary Material

Supplementary figure 1

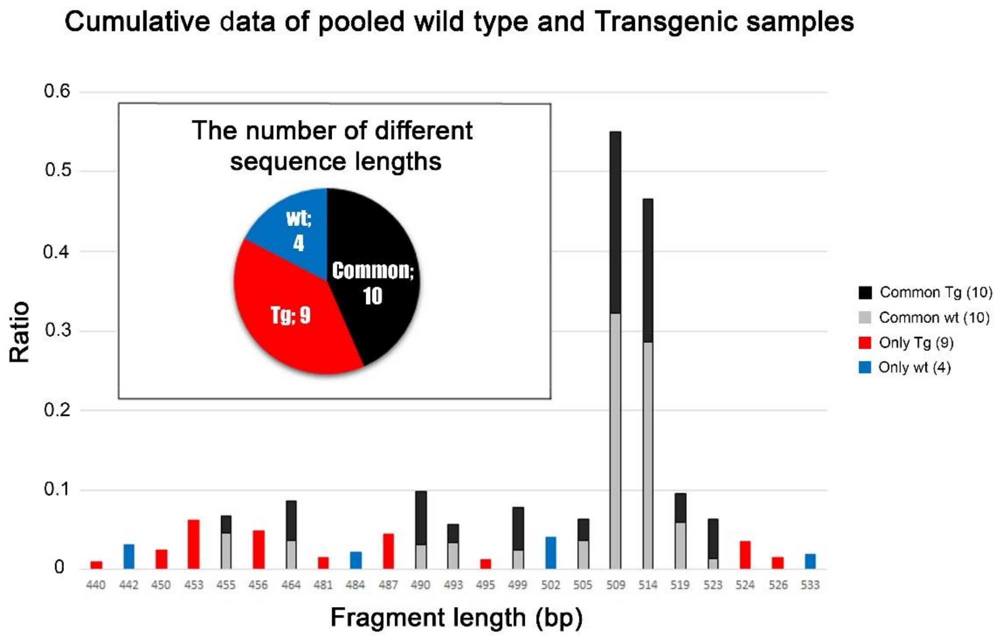

Length distribution analysis of variable regions and diversity measurement of Tg and wt mice. Length distribution analysis of Tg and wt mice after pooling the 4 Tg and the 4 wt samples at cDNA level. The Tg animals contained sequences with more distinct lengths (pie chart: 9 unique +10 common =19 Tg altogether vs. 4 unique +10 common =14 wt altogether (common: it was found in the wt and Tg samples as well) and their sequence length distribution was more even (bar chart). Sequence lengths unique to either wt or Tg mice are illustrated in blue and red, respectively.

Supplementary figure 2

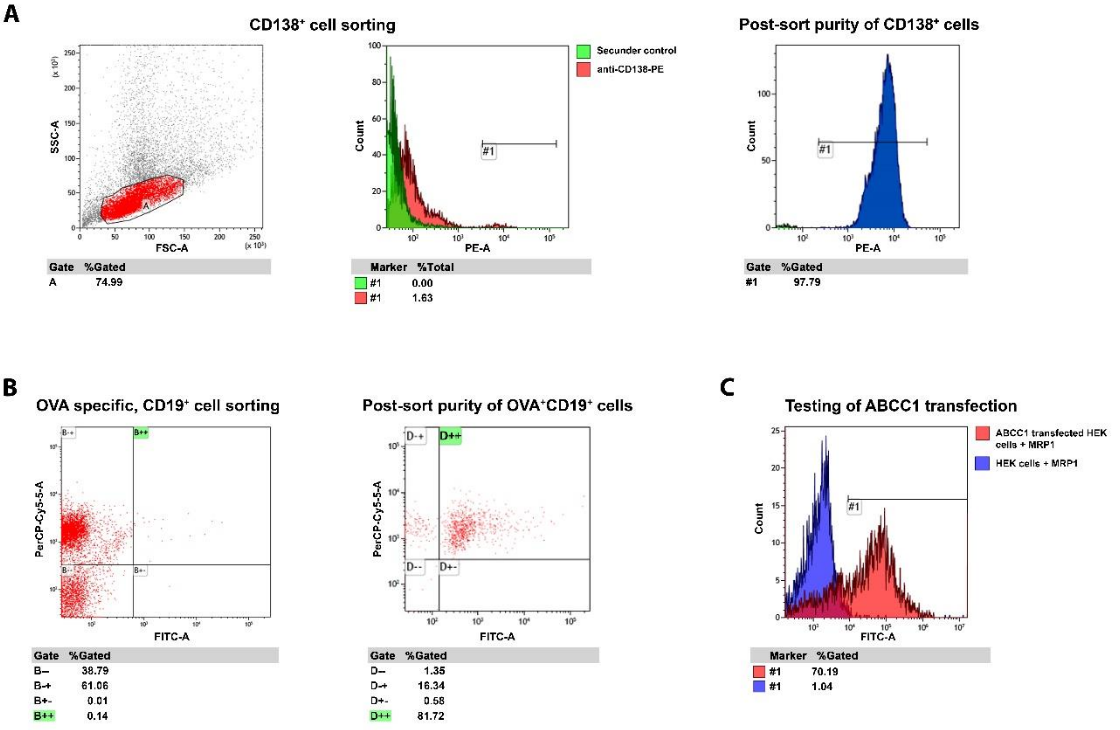

Representative graphs from different flow cytometry analyses. (A) CD138$^+$ cell sorting from an OVA immunized mouse and testing of the purity of the sorted cells. The cells in gate #1 were sorted out for further analysis (second figure). (B) CD19-PerCP-Cy5.5 and OVA-FITC double positive (or CD19$^+$OVA$^+$) cell sorting from an OVA immunized mouse and testing of the purity of the sorted cells. The cells in #B++ quadrat were sorted out for further analysis (first figure). (C) ABCC1 expression was tested in HEK cells using an antibody (MRP1, 50 times dilution) that can recognize the cytoplasmic region of the molecule.

Supplementary figure 3

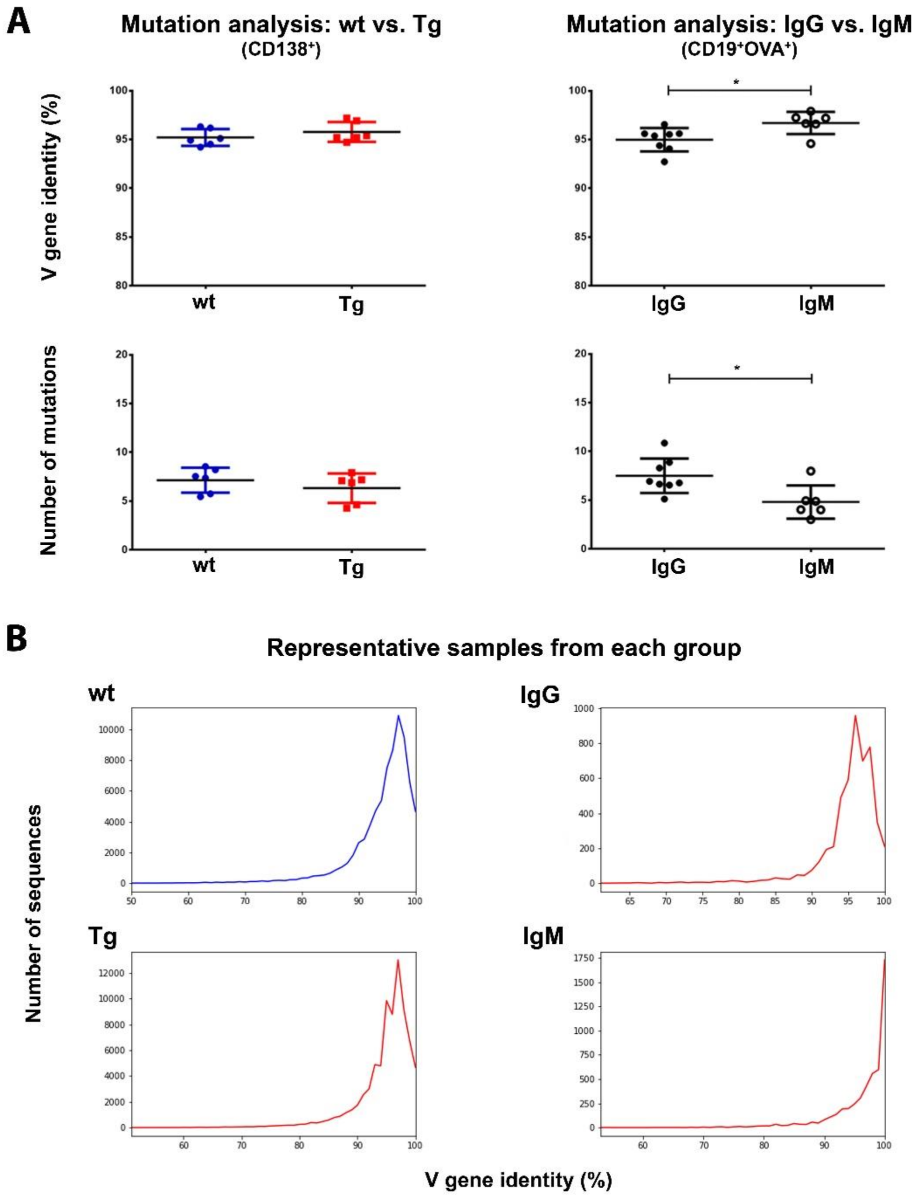

Analysis of V gene identity and mutation numbers after OVA immunization for Tg vs. wt animals () and on IgG vs. IgM level (). (A) The V gene identity shows how similar a given sequence is compared to the germline V gene sequence (100% means that there is no difference), while the number of mutations represent the exact number of the different nucleotides. Each individual point represents the average V gene identity or number of mutations of a given animal. In case of the IgG vs. IgM analysis, data derived form 8-8 mice (4-4 wt and 4-4 Tg) were used for comparison. Horizontal black lines and colored error bars show the mean ± SEM of the data (averaged over animals in the given group). Differences

between mean values were tested using unpaired t-tests. Statistically significant results are marked with asterisks (*: $p<0.05$). (B) The V gene identity of representative samples' repertoires of the investigated groups.

Supplementary figure 4

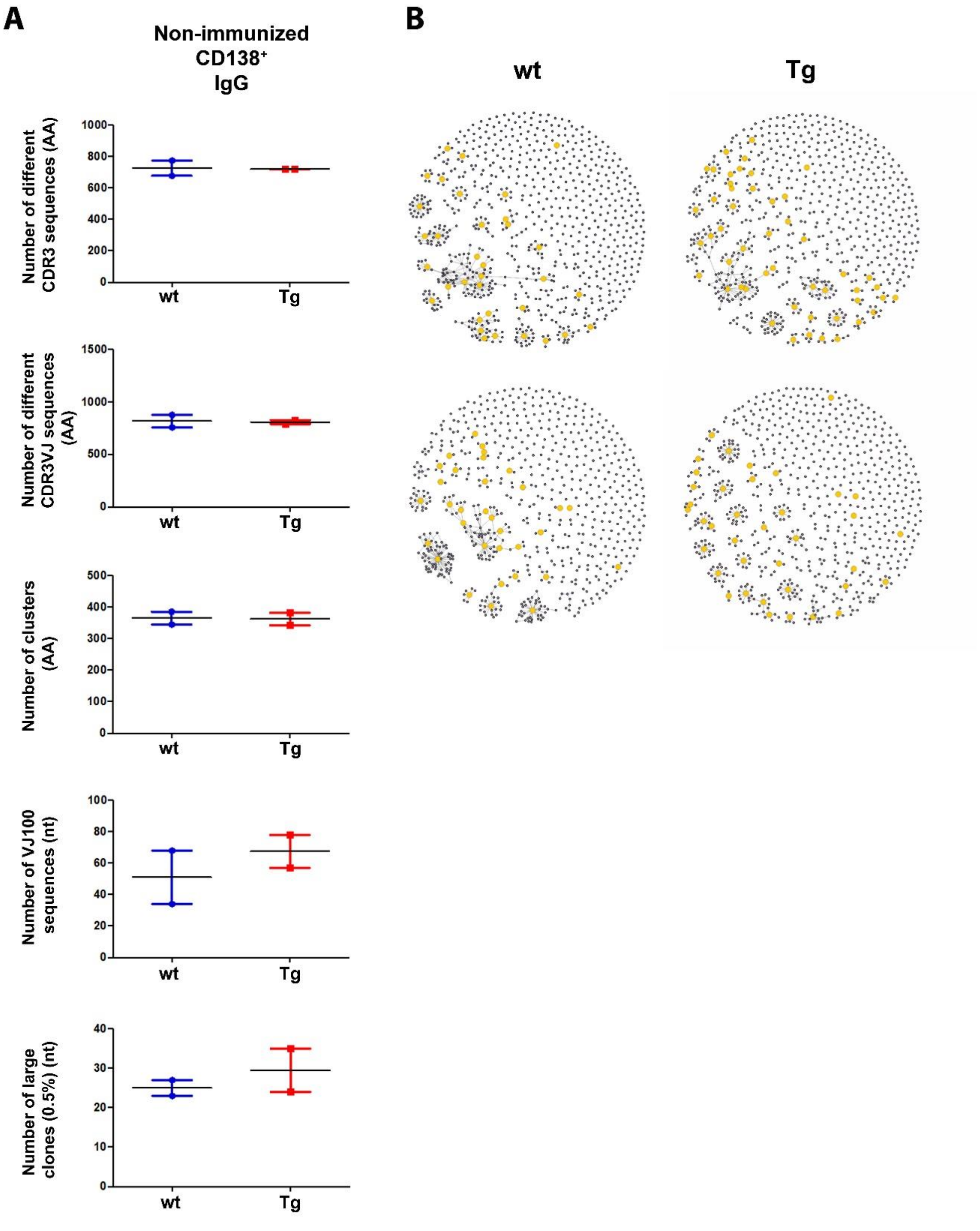

Diversity of the CD138$^+$ plasma cells of non-immunized animals. (A) Results for different diversity measures are plotted in each row. Horizontal black lines and colored error bars

represent the mean ± SEM of the data. Individual points correspond to specific animals. Due to the low number of mice in each group, no statistical analyses were carried out. (B) Network analysis of the non-immunized animals. Each vertex of the graph represents a single sequence with a link to those sequences that did not differ from it in more than one nucleotide. Vertex size corresponds to the number of identical sequences found. Yellow vertices represent highly expanded clones (harboring >0.5% of all sequences in the repertoire).

Supplementary figure 5

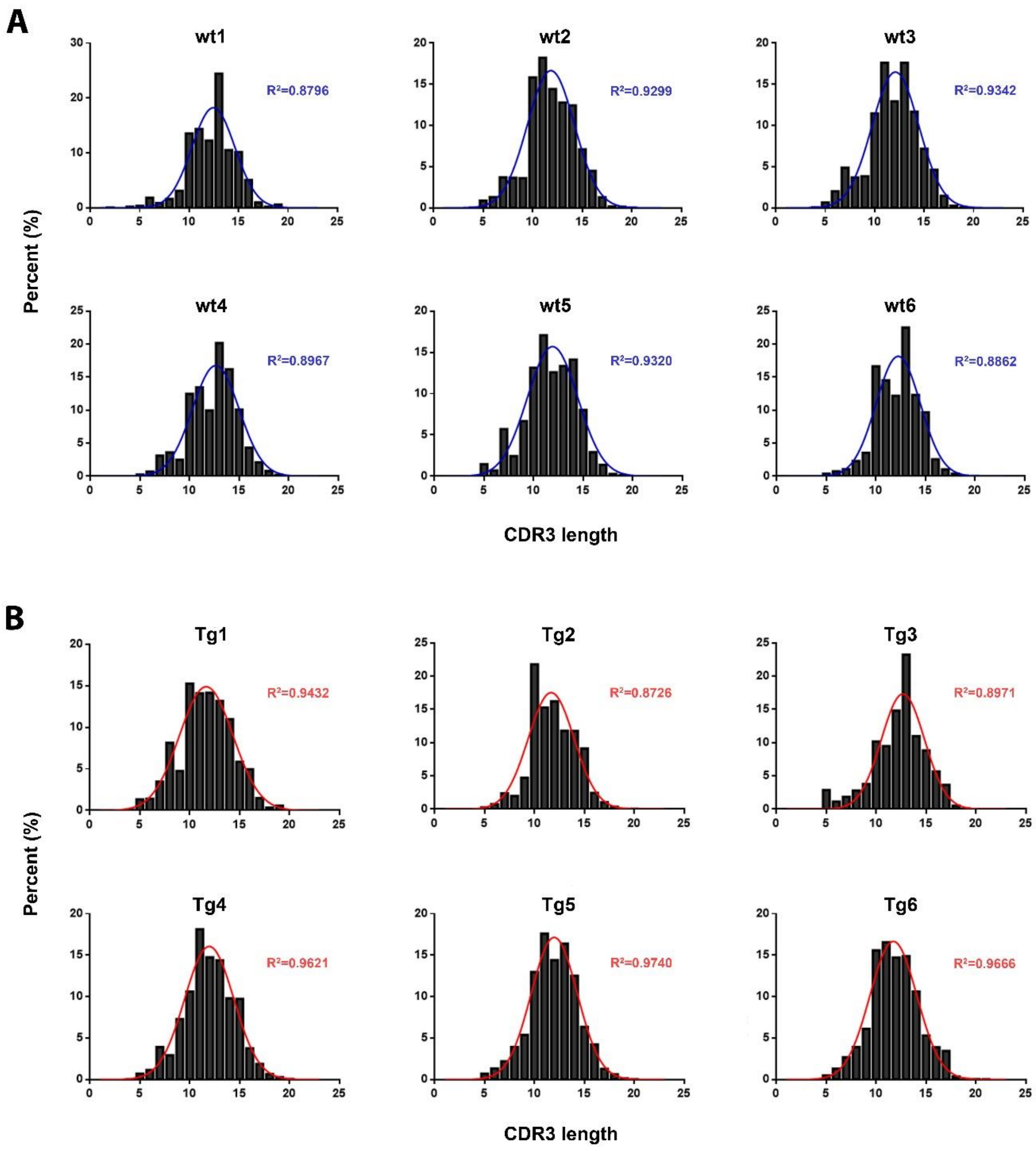

CDR3 length distribution of 6 wt (A) and 6 Tg (B) mice. The distribution of all samples follows a relatively normal (Gaussian) distribution with R^2-values (represents the goodness of fit) close to 1. The different mouse numbers refer to the different mice from the OVA immunization (experiment #3 in Table 2).

Supplementary figure 6

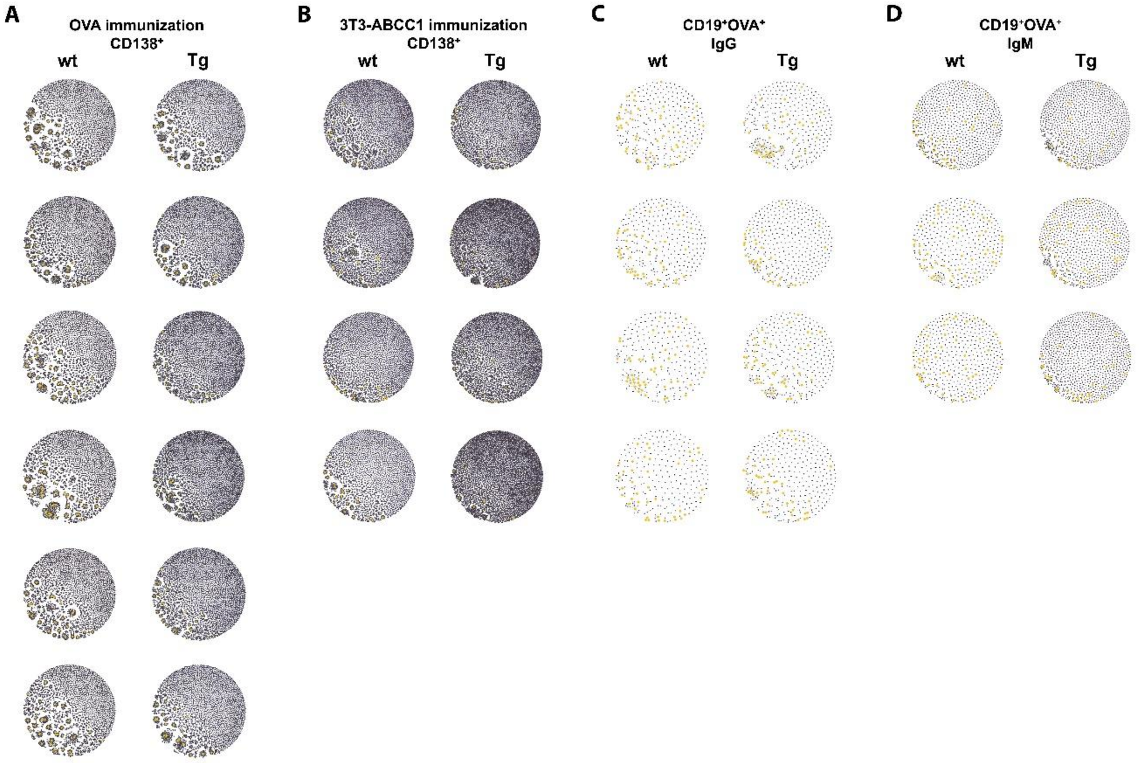

Network analyses of different samples. Network analysis of the CD138^{+} cells after OVA (A) or 3T3-ABCC1 (B) immunization. (C) CD19 and OVA double positive cells after OVA immunization at IgG level. (D) CD19 and OVA double positive cells after OVA immunization at IgM level. Each vertex of the graph represents a single sequence with a link to those sequences that did not differ from it in more than one nucleotide. Vertex size corresponds to the number of identical sequences found. Yellow vertices represent highly expanded clones (harboring >0.5% of all sequences in the repertoire).

Supplementary figure 7

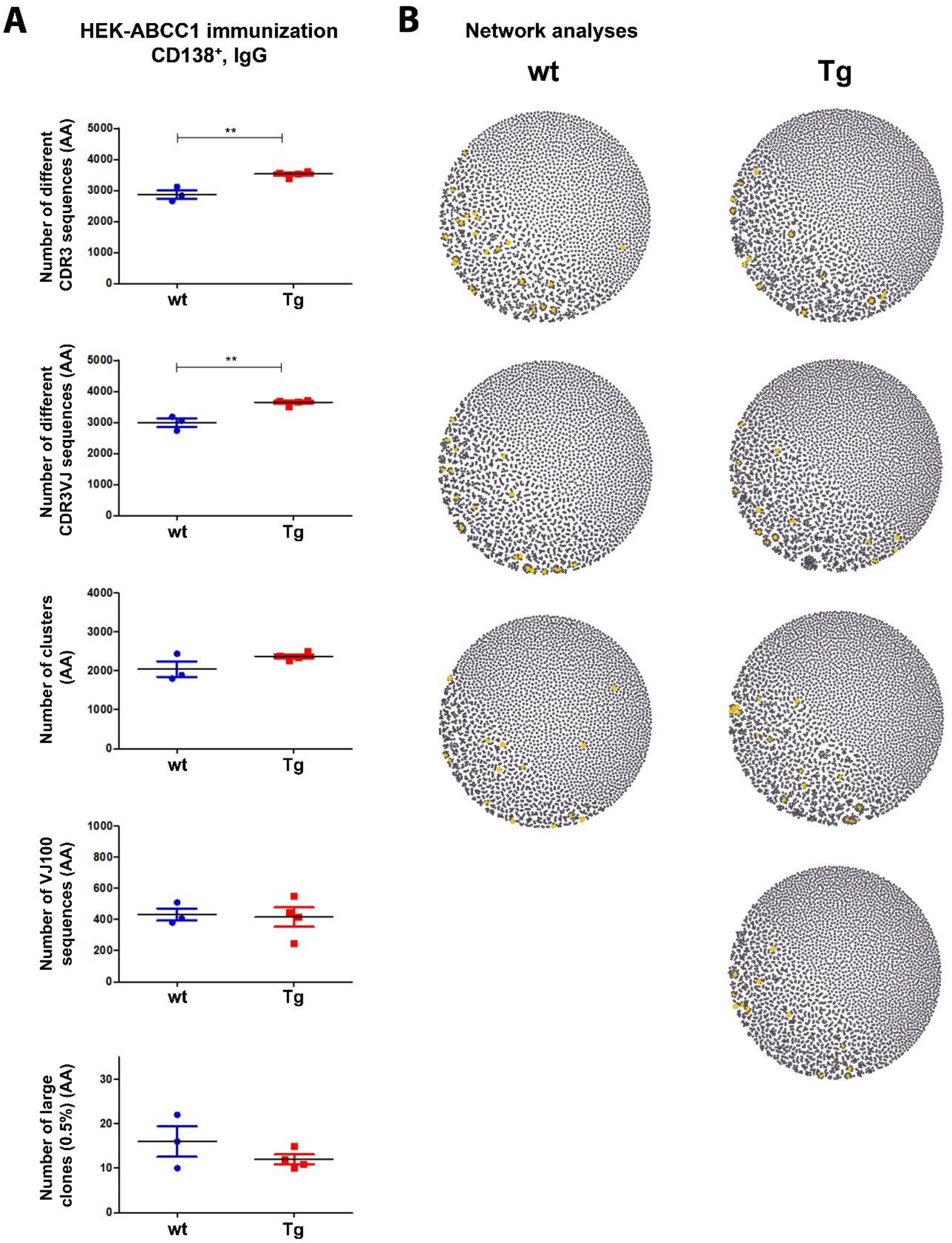

Diversity of the CD138⁺ plasma cells after HEK-ABCC1 immunization. (A) Results for different diversity measures are plotted in each row. Horizontal black lines and colored error

bars represent the mean ± SEM of the data. Individual points correspond to specific animals. Differences between mean values were tested using unpaired t-tests. Statistically significant results are marked with asterisks (**: $p<0.01$). (B) Network analysis for individual animals. Each vertex of the graph represents a single sequence with a link to those sequences that did not differ from it in more than one nucleotide. Vertex size corresponds to the number of identical sequences found. Yellow vertices represent highly expanded clones (harboring >0.5% of all sequences in the repertoire).

Supplementary figure 8

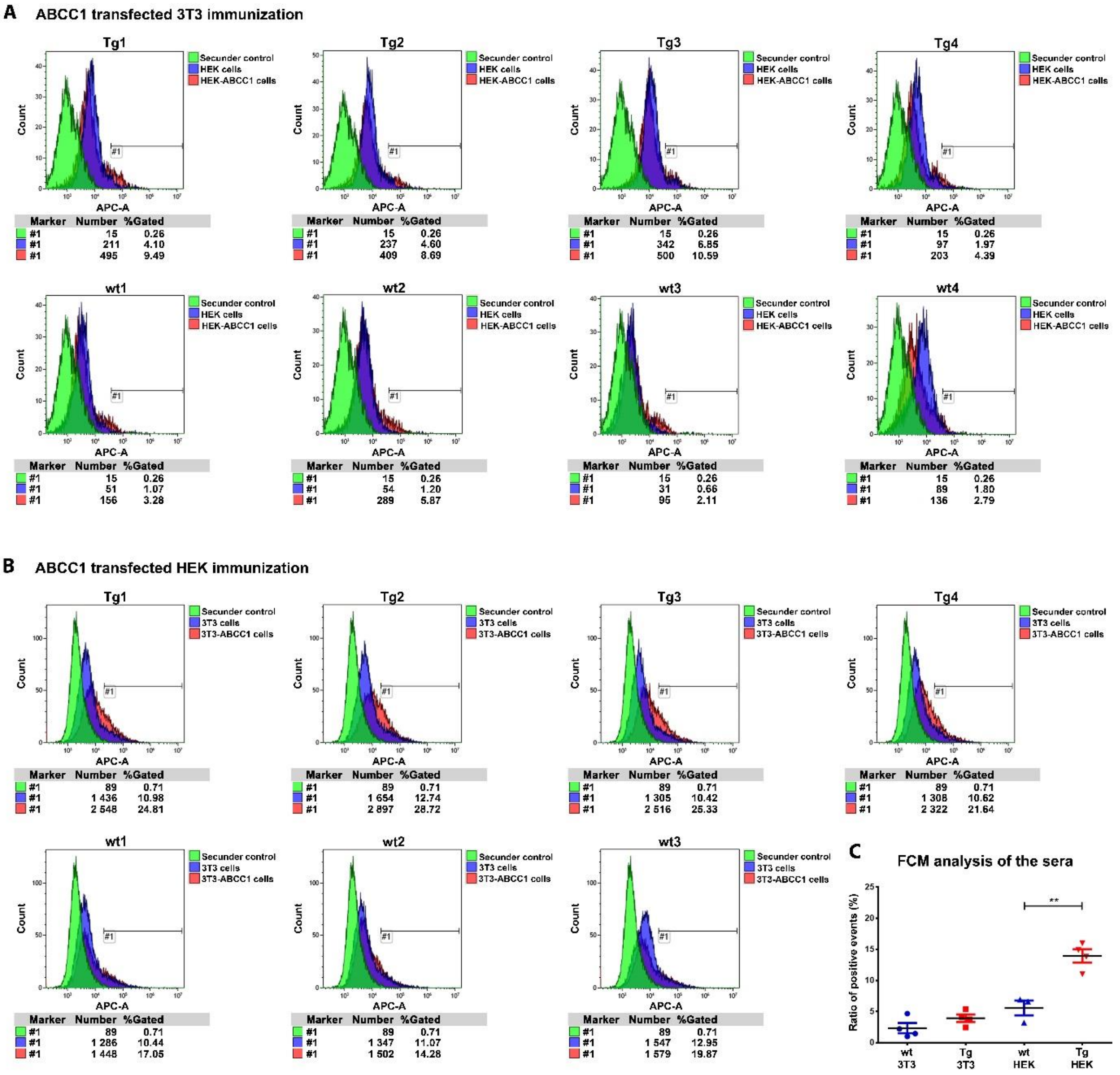

Flow cytometry analysis of the 3T3-ABCC1 and HEK-ABCC1 immunized mice sera. ABCC1 transfected HEK (A) or 3T3 (B) cells were used to detect the ABCC1 specific antibodies. The sera (1:1000 dilution, day 35) derived from the Tg animals recognized more transfected cells compared to wild type ones, especially in case of the HEK-ABCC1 immunization. (C) Ratios of the positive events for each animal group and immunization. Horizontal black lines and colored error bars represent the mean ± SEM of the data. Individual points correspond to specific animals. Differences between mean values were tested using unpaired t-tests. Statistically significant results are marked with asterisks (**: $p<0.01$). The

different mouse numbers refer to the different mice from the cellular immunizations (experiment #6 and #7 in Table 2).

Supplementary Table 1

Primers for NGS	Primer binding site	Sequence (5'-3')
**Reverse primer for cDNA synthesis (IgG)**	166 to 157	CAGGGATCCAKAGTTC
**Forward universal primer (PCR1)**	5' end of the adaptor sequence (RACE)	AAGCAGTGGTATCAACGCA
**Reverse primers (PCR1, IgG)**	149 to 143	AGGGAAATARCCCTTGACCAG
	149 to 143	AGGGAAGTAGCCTTTGACAAG
**Forward universal primer 2 (PCR2)**	5' end of the adaptor sequence (RACE)	CAGTGGTATCAACGCAGAG
**Reverse primers (PCR2, IgG)**	119 to 125	CARKGGATRRRCHGATGGGG
**Reverse primer (IgM)**	118 to 125	CCCAAGCTTACGAGGGGGAAGACATTTGGGAA

Summary of the primers used in the NGS experiments. The codon positions have been numbered according to Kabat numbering system. For the H chain, leader peptide is from codon position −20 to −1, framework region 1 is from 1 to 30, JH is from 100H to 113 and CH1 is from 114 to 223C. The primer may or may not completely span the first and the last codon of the primer binding site. [26]

Supplementary Table 2

Experiment	OVA immunization, CD138^{+} cells, 77.000 analyzed sequences				
**Animals**	**CDR3 clones**	**CDR3VJ clones**	**VJ100 sequences**	**Number of clusters**	**Number of large clones (>0.5%)**
wt	2900	3596	556	1541	23
	2559	3080	450	1181	28
	3050	3644	424	1760	18
	2530	3182	310	1134	32
	2391	3123	537	1167	26
	2655	3247	322	1327	25
Tg	2990	3645	667	1595	19
	3426	4422	2401	2284	18
	3955	4669	1262	2708	17
	2950	3745	865	1833	22
	3538	4185	982	2190	21
	4362	5364	1051	2819	22
**Experiment**	**Non-immunized, CD138^{+} cells, 16.780 analyzed sequences**				
**Animals**	**CDR3 clones**	**CDR3VJ clones**	**VJ100 sequences**	**Number of clusters**	**Number of large clones (>0.5%)**
wt	775	877	34	386	27
	678	759	68	345	23
Tg	721	791	57	383	35
	720	828	78	343	24
**Experiment**	**OVA immunization, OVA^{+}, CD19^{+} cells, 8.000 analyzed sequences, IgM**				
**Animals**	**CDR3 clones**	**CDR3VJ clones**	**VJ100 sequences**	**Number of clusters**	**Number of large clones (>0.5%)**
wt	812	822	409	707	21
	394	395	257	339	28
	453	460	187	411	17
Tg	1065	1087	497	921	7
	789	798	464	687	16
	1039	1067	347	930	17
**Experiment**	**OVA immunization, OVA^{+}, CD19^{+} cells, 5.000 analyzed sequences, IgG**				
**Animals**	**CDR3 clones**	**CDR3VJ clones**	**VJ100 sequences**	**Number of clusters**	**Number of large clones (>0.5%)**
wt	298	324	44	186	36
	308	308	47	214	24
	258	274	43	169	28
	271	276	36	205	17
Tg	358	372	38	217	24
	382	389	39	315	2
	341	365	37	210	30
	341	349	39	248	26
**Experiment**	**3T3-ABCC1 immunization, CD138^{+} cells, 85.000 analyzed sequences**				

Animals	CDR3 clones	CDR3VJ clones	VJ100 sequences	Number of clusters	Number of large clones (>0.5%)
wt	4024	4575	505	2316	18
	4293	4861	743	2561	16
	3488	3946	1144	2409	17
	2897	3340	1157	1869	21
Tg	3626	3831	1336	2549	13
	5708	6235	1284	3880	12
	4635	5214	1063	2848	14
	5862	6345	1444	4063	11
**Experiment**	**HEK-ABCC1 immunization, $CD138^+$ cells, 42.000 analyzed sequences**				
**Animals**	**CDR3 clones**	**CDR3VJ clones**	**VJ100 sequences**	**Number of clusters**	**Number of large clones (>0.5%)**
wt	2659	2736	381	1805	22
	2836	3050	408	1894	16
	3120	3182	510	2445	10
Tg	3393	3513	249	2274	10
	3572	3719	445	2385	12
	3544	3661	417	2342	15
	3616	3695	553	2512	11

Summary of the different datasets obtained by NGS analyses.
